# Congenital pulmonary hypoplasia combined with congenital cardiac disease and ectopic kidney: a case report

**DOI:** 10.2478/abm-2023-0066

**Published:** 2023-10-26

**Authors:** Ling Lu, Sujuan Hu, Gaoyan Wang, Rong Jin, Renzheng Guan, Fengjing Cui, Zhenghai Qu, Dongyun Liu

**Affiliations:** 1 Department of Pediatrics, The Affiliated Hospital of Qingdao University, Qingdao 266003, China

**Keywords:** abnormalities, bronchi, congenital, hypoplasia, lung, prognosis

## Abstract

**Background:**

Congenital pulmonary hypoplasia (CPH) is a rare pulmonary disease featured by incomplete development of pulmonary tissues. Its diagnosis is still a challenge as patients are usually misdiagnosed as atelectasis.

**Case presentation:**

A female neonate was admitted to our hospital due to post-birth jaundice for 12 hrs. Physical examination showed accelerated breathing. There was no respiratory sound in the left lung. Chest film indicated decline of lucency in the left lung. Chest CT scan indicated absence of left lung and primary bronchus of the left lung. The boundary between left mediastinum was not clearly displayed. Three-dimensional CT scan indicated absence of left lung and left principal bronchus. Cardiac ultrasonography confirmed congenital heart disease. She showed ectopic kidney. Finally, she was diagnosed with CPH concurrent with congenital heart disease and ectopic kidney.

**Conclusions:**

On 17-month follow-up visit, the patient is still survived, but she presents with obstruction in ventilation function.

Congenital pulmonary hypoplasia (CPH) is a rare type of pulmonary disease involving congenital malformation in lung, bronchus and pulmonary vessels induced by fetal maldevelopment [1]. In clinical practice, there is still misdiagnosis in the neonatal CPH, especially those with mild symptoms. Also, patients with severe CPH are usually misdiagnosed as atelectasis, causing treatment delay. We presented a rare neonatal CPH patient concurrent with congenital heart disease and ectopic kidney, and discussed the pathogenesis, classification, diagnosis, treatment and prognosis.

This study was approved by the Ethics Committee of Affiliated Hospital of Qingdao University (certificate of approval No. QYFYWZLL26558). The authors have obtained written informed consent from the patient's parents.

## Case presentation

A female neonate with a birth weight of 2,250 g was admitted to our department on March 18, 2019 due to post-birth jaundice for 12 hrs. She was born at a gestational age of 37 week and 3 days via spontaneous delivery. APGAR score was 10. Her body length (L) was 47 cm and her head circumference (HC) was 33 cm (ponderal index = 2.17, L/HC = 1.42), belonging to symmetrical type of small for gestational age (SGA) roughly [2]. She has no facial dysmorphic feature. There was no intra-uterine stress.

**Figure 1.**
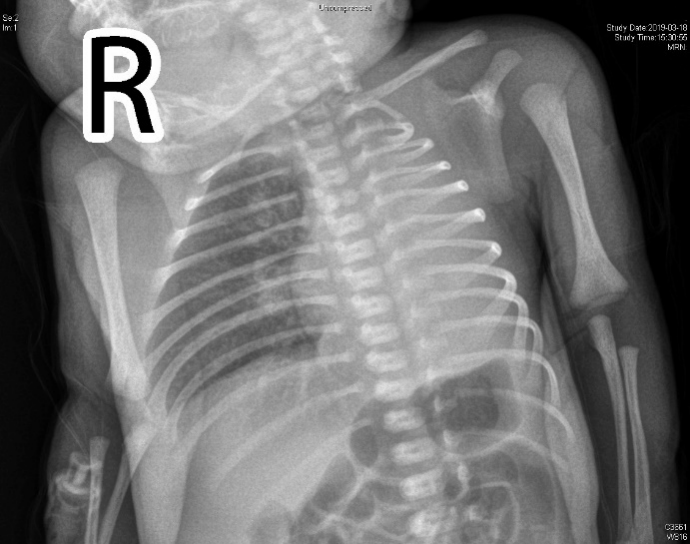
Bedside chest film indicated lucency decline in the left lung. The pulmonary markings in the right lung were vague.

**Figure 2.**
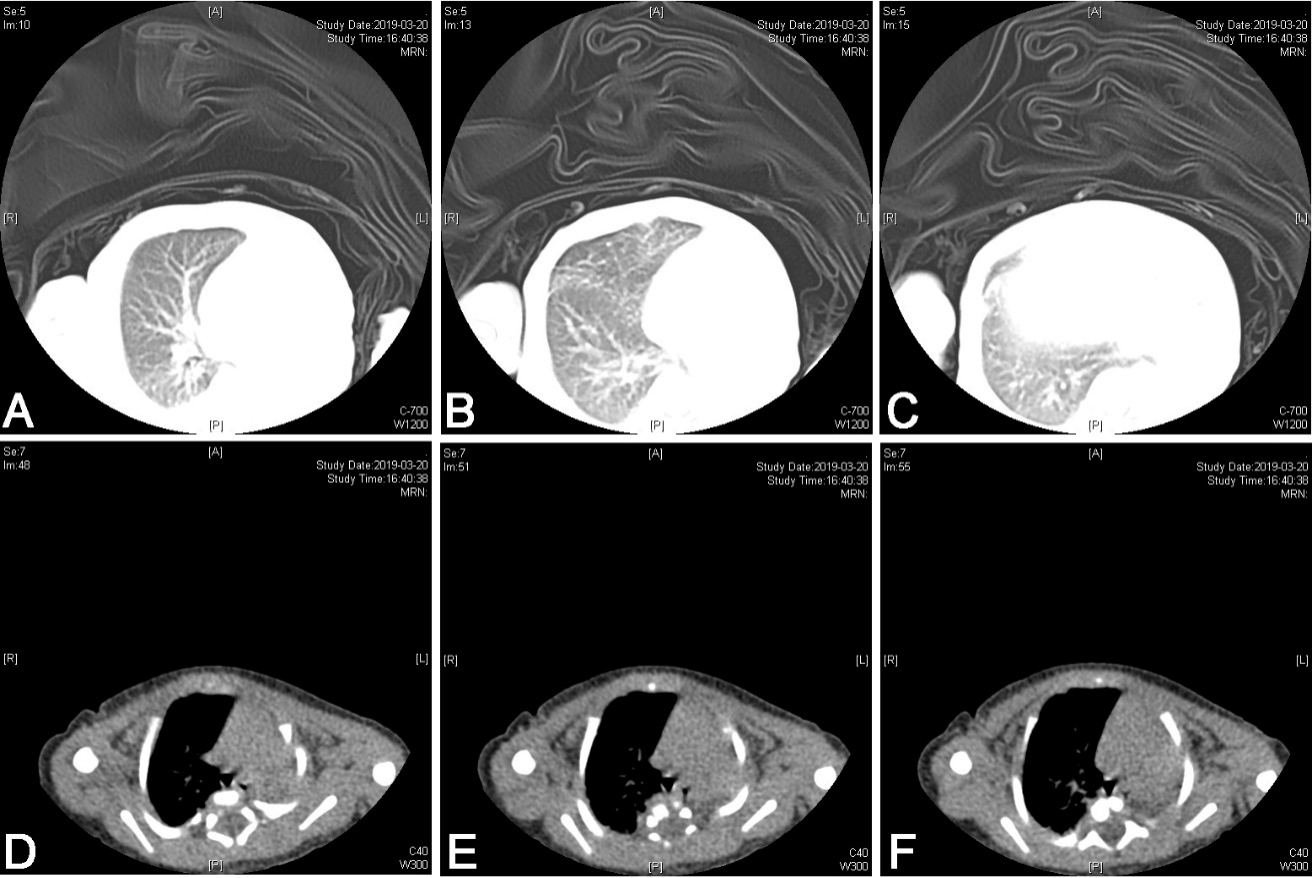
Thoracic CT indicated that the left lung and the primary bronchus of left lung were not available in pulmonary window. No shadows with aberrant densities were noticed in the right lung **(A–C)**. In addition, there was mediastinal shift to left side in mediastinal window. The structures and boundaries in left mediastinum were not completely displayed **(D–F)**.

**Figure 3.**
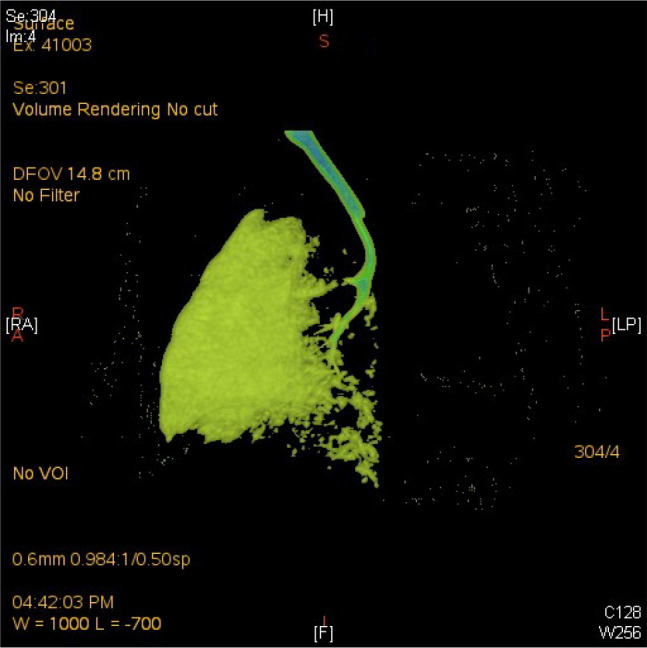
Three-dimentional imaging indicated that trachea, right principal bronchus and three bronchus reached the three lobes of the right lung. The left lung and left principal bronchus were not available.

As she showed accelerated breathing, a conventional physical examination was given. There was no respiratory sound in the left lung according to auscultation. Chest film indicated decline in lucency of left lung (**Figure 1**). Thoracic CT scan was recommended as the size of reduced pulmonary lucency in chest film was too large (**Figure 2**). On day 3, thoracic CT and pulmonary window indicated absence of left lung and primary bronchus in the left lung. For mediastinal window, there was mediastinal shift to the left side. Structural boundary within left mediastinum was not completely displayed. Three-dimensional imaging indicated extension of trachea, right principal bronchus and bronchus into right lung lobe, while the left lung and left principal bronchus were not visualized (**Figure 3**). Cardiac ultrasonography confirmed congenital heart disease presenting ventricular septal defect (VSD, 0.18 cm), patent ductus arteriosus (PDA, 0.3 cm × 0.2 cm), patent foramen ovale (0.28 cm), pulmonary hypertension (43 mmHg), mild to moderate tricuspid regurgitation, as well as slight pericardial effusion. Moreover, she showed ectopic kidney. Finally, she was diagnosed with CPH combined with congenital cardiac disease and ectopic kidney.

As mentioned above, the child was diagnosed with symmetrical SGA, which occurred mostly in early pregnancy and was associated with some diseases that seriously affected the number of fetal cells and the growth potential was often reduced. In addition to genetic factors, other factors such as infection or severe maternal hypertension can also contribute to reduced fetal growth potential. Virus infection in the first trimester of pregnancy has the most serious consequences and can significantly affect cell replication and birth weight. Genetic defects and chromosomal abnormalities can also cause growth restriction in early pregnancy [2]. Her mother was healthy during pregnancy with no intrauterine virus infection. The parents were in good health with no family history of genetic diseases. Therefore, we speculate that the reason of SGA is likely related to twin pregnancy.

She was a survivor of monozygotic twin pregnancy, and the other twin developed abnormally at 11 weeks gestational age and stopped developing at 14 weeks gestational age. Her mother showed a high risk of Down syndrome in the prenatal screening at a gestation age of 16 weeks, but no further examination and genetic tests were performed as they finally refused.

**Figure 4.**
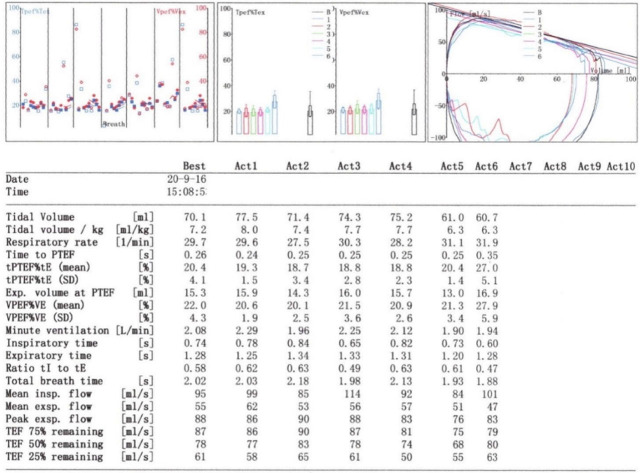
Pulmonary function test indicated that the patient was considered to present obstructive ventilation dysfunction. The tidal volume was normal (7.2 ml/kg). Time to peak tidal expiratory flow as a proportion of expiratory time [TPTEF/TE(%)] was 20.4% (normal range: 28%–55%), and volume to peak expiratory flow as a proportion of exhaled volume [VPEF/VE (%)] was 22% (normal range: 28%–55%).

For the treatment, venous pumping of Piperacillin and Tazobactam (50 mg/kg, q12h) and Ambroxol (15 mg, q12h) were given for 5 days. On the follow-up visit, her parents showed poor treatment compliance. About 9 months after delivery, she showed cough and wheeze combined with low fever. Besides, there was wheezy phlegm and wheezing sound in the right lung, and symptoms were attenuated after anti-infection therapy and atomization. At 17 months old, she was recommended to receive pulmonary function determination in our hospital, together with tidal breathing lung function test (**Figure 4**). The tidal volume was normal (7.2 ml/kg). Time to peak tidal expiratory flow as a proportion of expiratory time [TPTEF/TE(%)] was 20.4% (normal range: 28%–55%), and volume to peak expiratory flow as a proportion of exhaled volume [VPEF/VE (%)] was 22% (normal range: 28%–55%). On this basis, obstructive dysfunction of pulmonary ventilation was considered.

## Discussion

CPH refers to malformation of lung, bronchus and pulmonary vessels at the embryonic stage [3]. Most CPH is induced by secondary causes, especially those with CPH induced by space-occupying lesions in thoracic cavity such as congenital diaphragmatic hernia, a giant esophageal hiatal hernia, congenital cystic adenomatoid malformation of lung, bronchial cyst, pulmonary sequestration, and teratoma [4].

CPH is divided into three categories according to Boyden classification, including pulmonary agenesis, pulmonary aplasia and pulmonary hypoplasia [5]. In this case, the patient was classified into pulmonary agenesis with involvement of the left lung. Unfortunately, most neonates with malformation in both lungs and those with no pulmonary development would not show a long-time survival [6]. Indeed, patients with single side pulmonary agenesis and pulmonary aplasia may survive in the presence of good compensation function [7]. However, these patients may combine with other severe malformation such as congenital cardiac disease, tracheo-asophageal atresia, spinal cord deformity, and severe renal dysfunction. Usually, these symptoms are severe leading to dyspnea, refractory respiratory infection and feeding problem. Therefore, about 50% of the cases were not survived at the infant stage [8, 9]. For cases with pulmonary hypoplasia, disease severity was associated with malformation and concurrent malformations. Patients of a mild severity may not present symptoms at infant stage, but they may present refractory respiratory infection and disease progression. These with severe conditions may present dyspnea, cyanosis and respiratory failure, together with decline in respiratory movement and respiratory sound, as well as cardiac sound shifting to the involved side. In this case, the patient was admitted to a local hospital due to cough and wheeze and survived with no anormaly in growth and development at the age of 17 months. Thus, close attention should be paid to the recurrence of refractory respiratory infection as it may affect the outcome.

Imaging findings including thoracic X ray, CT scan and contrast and enhanced CT scan, angiography, MRA, bronchoscope and pulmonary function could contribute to the diagnosis. The typical cases could be confirmed through thoracic X ray which was manifested as a small size in involved lung, decline in pulmonary marking, elevation of diaphragm, and deviation of mediastinum to involved sites. CPH patients are usually manifested as mixed ventilation dysfunction, elevation in airway resistance, and decline of compliance. Those with mild pulmonary mal-development may present normal pulmonary function, however, they may present pulmonary hyperinflation in distal end and obstructive pulmonary ventilation dysfunction in the presence of primary bronchial stenosis or refractory infection induced bronchial stenosis. Our case showed obstructive respiration disorder at 17 months old, which predicted a poor prognosis in the future.

Close attention should be paid to distinguish CPH from neonatal pneumonia, pulmonary atelectasis induced by foreign body inhalation, and bronchial asthma. Our case showed accelerated breathing after birth, together with lucency decline in the left lung. Then lobar pneumonia was considered in the left lung. Clinical manifestations and chest X ray findings of atelectasis due to foreign body in bronchus were similar with those caused by pulmonary hypoplasia. Nevertheless, presence of foreign bodies in bronchus may present a sharp onset in those with a history of foreign body inhalation. Thoracic X ray and bronchoscope may further contribute to its diagnosis. Mild pulmonary hypoplasia children should be identified from those with bronchial asthma. Those with mild pulmonary hypoplasia would present refractory bronchial and pulmonary infection, together with refractory gasping. The symptoms of children with bronchial asthma could be attenuated mainly after the relieving cough and asthma, whereas, there might be progression in children with pulmonary hypoplasia. The course of children with pulmonary hypoplasia was prolonged, and their condition was even serious. If this child had not been hospitalized for jaundice after birth, it was difficult to screen the left lung hypoplasia. She should be diagnosed with bronchial asthma in cases of recurrent wheezing, combing with obstructive ventilation dysfunction. In clinical practice, CPH treatment is mainly relied on symptomatic treatment. Inhalation of oxygen was given in cases of hypoxemia and mechanic ventilation should be given in the presence of severe conditions.

Pulmonary hypoplasia was concurrent with some congenital diseases, but there were rare CPH cases except one article published in 1982, in which Helms et al [10] reported 19 CPH aged 2–8 weeks, including 9 with congenital diaphragmatic hernia, 5 with Werdnig-Hoffman disease of intrauterine onset, and 5 with isolated pulmonary hypoplasia. However, there is a lack of treatment data and follow-up data in that study. In a long-term follow-up of 18 children with pulmonary hypoplasia (median age:10.5 months), Gursoy et al [11] found that the main cause of hospitalization was pneumonia, and that the age of diagnosis was positively correlated with the number of cases of pneumonia. After surgery, 3 of the 9 children had thoracic malformations, 2 had scoliosis, and 2 had diaphragmatic eminence.

## Conclusion

We presented a rare neonatal CPH patient combined with congenital heart disease and ectopic kidney, and discussed its differential diagnosis and treatment. The patient is still alive in the 17-month follow-up with obstruction in ventilation function. CPH patients are mainly manifested as mediastinal shift, narrowing in intercostal space, and absence of respiratory sound. The key point for treating CPH is to attenuate the pulmonary fibrosis induced by refractory infection, in order to improve quality of life and extend life span.
